# The epidemiology of suicidal behaviors among the countries of the South Asia: A systematic review and meta analysis

**DOI:** 10.12669/pjms.41.6.12041

**Published:** 2025-06

**Authors:** Nazish Imran, Ahmed Waqas, Sania Mumtaz Tahir, Roop Kiran Khan, Maryam Ayub, Hareem Arshad, Bariah Rafiq, Sadiq Naveed

**Affiliations:** 1 Nazish Imran, FRCPsych, MRCPsych, MHPE, PhD. Professor, Head Department of Child & Family Psychiatry, King Edward Medical University, Lahore, Pakistan; 2 Ahmed Waqas, PhD. Institute of Population Health, University of Liverpool, Liverpool, UK; 3 Sania Mumtaz Tahir, MBBS, FCPS, Fellow. Child & Family Psychiatry Department, King Edward Medical University, Lahore, Pakistan; 4 Roop Kiran khan, MBBS, FCPS. Senior Registrar, Academic Department of Psychiatry and Behavioral Sciences, King Edward Medical University, Lahore, Pakistan; 5 Maryam Ayub, FCPS, MRCPsych, Fellow. Child & Family Psychiatry Department, King Edward Medical University, Lahore, Pakistan; 6 Hareem Arshad, MD Resident Physician, Penn State Health Milton S. Hershey Medical Center, Hershey, PA; 7 Bariah Rafiq, MBBS Senior House Officer, Dublin North City Mental Health Services, Ireland; 8 Sadiq Naveed, MD, MPH Psychi atry Program Director, Eastern Connecticut Health Network, USA

**Keywords:** Suicide, Suicidal ideation, Suicide attempts, Non suicidal behaviors, South Asia, Suicidal behavior, Systematic review, Meta-analysis

## Abstract

Despite the alarming suicide burden, South Asia lacks sufficient literature and research. The objective of this systematic review and meta-analysis was to appraise the current evidence and estimate the prevalence of suicidal behaviors (ideation, plan, attempts, completed suicide and non-suicidal self-injury (NSSI)) among countries in South Asia. We systematically searched PubMed, PsycINFO, Web of Science, Scopus, and CINAHL for observational and longitudinal studies involving statistical analysis of suicidal behaviors in south Asian countries as per the PRISMA guidelines, without any limitations concerning age, gender, language, or year of publication.

A random-effects model was used to synthesize prevalence data, and mixed-effects regression models were employed for subgroup analyses. Main outcome was lifetime, period and point prevalence of completed suicide, suicidal ideation, plan, and Non suicidal behaviors. A total of 27 studies representing data pertaining to suicidality and related behaviors in South Asian countries were included. The point prevalence of suicidal ideation was 16.7% (95% CI: 11.5% to 23.5%), suicidal plan was 13.6% (13.0% to 14.2%) and attempted suicide was 5.6% (95% CI: 3.5% - 9.0%). The 12-month period prevalence for suicidal ideation was noted to be 11.8% (95% CI: 5.5% to 23.6%), plan 3.6% (95% CI: 2.1% to 6.0%). Suicidal attempts 3.0% (95% CI: 1.6% to 5.5%), completed suicides 0.1% (95% CI: 0% to 0.8%) and non-suicidal behaviors 6.5% (95% CI: 0.7% to 39.5%).

Lifetime prevalence ranged from suicidal thoughts 15.9% (95% CI:15.0% to 16.8%), suicidal plan 7.9% (95% CI: 6.2% to 10.1%), attempts 3.1% (95% CI: 1.2% to 7.8%) and non-suicidal behaviors 44.8% (95% CI: 41.2% to 48.4%). We found evidence of significant heterogeneity for suicidal behaviors in the studies included. The epidemiological burden of suicidal behaviors among countries in South Asia appears to be very high. Knowledge about the epidemiology of such behaviors is important for policymaking and to inform context specific interventions to reduce loss of lives caused by suicidal behaviors.

## INTRODUCTION

Suicide is a significant global public health concern, claiming over 700,000 lives annually, with 1.3% of all deaths in 2019 attributed to suicide.^1^,^2^ In young individuals aged 15-19, it is the fourth leading cause of death globally.^1^ Suicide trends vary across the world owing to the diversity of culture, religion context and environment. Although suicides occur worldwide, most deaths by suicide (77%) occur in low- and middle-income countries.^1^

South Asian countries (India, Pakistan, Nepal, Sri Lanka, Bhutan, Bangladesh, Afghanistan, and the Maldives) comprises 25% of the world’s population, exhibit high rates of common mental disorders, particularly in the 15-29 age group, where suicide is a leading cause of death.^3^-^5^ Epidemiological patterns in South Asian region are diverse and different from West; some notable factors which need mention are different gender distribution ratio, more pronounced stigma and different factors related to sociocultural fabric.^6^ Suicide is multifactorial and results from the interplay of various factors and among them mental illnesses constitute a major factor. Psychological autopsy studies indicate that 90% of people who died by suicide had mental illness.^6^ Among common mental disorders, depression is most closely linked with high suicide rates. Arafat et al (2022) reported in their systematic review that the prevalence of depression was as high as 78% in non-fatal suicide attempts and up to 79% in suicides in South Asia.^7^

WHO estimated suicide rates in South-East Asia (10.2 per 100,000) region are much higher than the global average (9.0 per 100,000) in 2019.^1^ Furthermore, the South-East Asia (SEA) region had a much higher female age-standardized suicide rate (8.1 per 100,000) compared to the global female average (5.4 per 100,000).^1^ Mazumder (2022) in their systematic review focusing on suicide in women in South Asia reported higher suicide prevalence rates in females in South Asia as compared to European countries due to vulnerability factors. Furthermore, the prevalence of suicidal ideation was 17% and suicide attempts as 5%, while attempt were more common in adolescents and ideation more in adult women.^8^ Pesticide poisoning is the most common suicide method in both rural and urban populations in South Asia.^9^ A recent meta-analysis of suicide estimates in adolescent population found pooled prevalence of suicidal ideation to be 11% (95 % CI 7–15); while suicidal attempts and suicidal plan was estimated to be 3 % each (95 % CI 2–5).^10^

There are concerns that suicide rates in South Asia are even much higher than officially reported due to under reporting of suicide because of various reasons. Lack of national suicide registries, under reporting of suicide and suicidal behaviors due to suicide being considered shameful as well as it being a criminal act in some countries with punishments including jail terms and financial penalties has led to questioning of the validity of the data officially reported by countries of the region to WHO.^2^ Despite the high burden of suicide in South Asia, this region lags behind in the reporting and adequate data for suicide.^9^ United Nations Sustainable Development Goals (SDGs) and WHO’s Comprehensive Mental Health Action Plan 2013– 2030 aims to reduce the global suicide mortality rate by one third by 2030.^1^ However, understanding of the epidemiology of suicidal behaviors, which forms the backbone of effective suicide prevention strategies is lacking in South Asian region. The objective of this systematic review and meta-analysis was to appraise the current evidence and estimate the prevalence of suicidal behaviors (ideation, plan, attempts, completed suicide and non-suicidal self-injury (NSSI)) among countries in South Asia.

## METHODS

**Fig.1:**
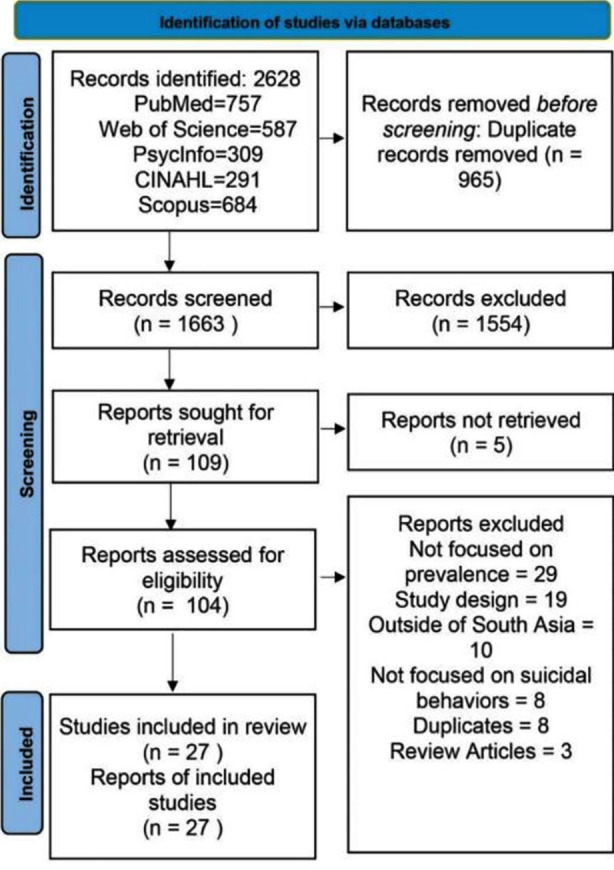
Prisma Flow Diagram.

Systematic Review was conducted as per the Preferred Reporting Items for Systematic Reviews and Meta-Analyses (PRISMA) guidelines.^11^ (Fig.1) A comprehensive search of several databases, including PubMed, PsycINFO, Web of Science, Scopus, and CINAHL, was executed using these search terms; (“Prevalence” OR “Frequency” OR “epidemiology*” OR “epidemiological” OR “proportion”) AND (“suicide” OR “commit suicide” OR “attempt suicide* attempt” OR “completed Suicide” OR “plan suicide” OR “suicide idea” OR “suicide behavior” OR “self-harm” OR “parasuicide” OR “selfpoison*” OR “Self-Poison*” OR “self-injury” OR “Self-Injurious Behavior”) AND (“South Asia” OR “Afghanistan” OR “Nepal” OR “Pakistan” OR “India” OR “Sri Lanka” OR “Bhutan” OR “Bangladesh” OR “Maldives”). No limitations were set concerning age, gender, language, or year of publication. For examining the prevalence of suicidal behaviors, observational studies and initial evaluations from longitudinal research conducted in a South Asian country having more than 400 sample size and random sampling were considered. The focus was on the population ranging from children to adults across South Asia. Studies involving statistical analyses of suicidal behaviors from any South Asian country were included, without language, age, or publication date restrictions. Exclusion criteria consisted of book chapters, conference papers, abstract-only articles, case reports, or opinion pieces, as well as studies with overlapping data sets. The primary outcomes investigated were the prevalence rates of different suicidal behaviors in South Asian countries. Different teams of two independent reviewers (SN, NI, AW, SMT, RKK, MA, BR and HA) conducted article screening and data extraction, resolving any disagreements through discussion. Quality assessment was done using adapted Newcastle-Ottawa scales for different study types.^12^ Two reviewers (SMT, MA) independently evaluated the quality of studies without binding to author or journal information. Discrepancies in decision making were then resolved by discussion with two senior authors (NI, SN).

The quality of the studies was measured using Newcastle-Ottawa scales across seven matrices: representativeness of the population, adequacy of sample size, percentage of respondents, quality of tools used for the ascertainment of mental disorder, presence of sub-group analysis, quality of method used for assessment of outcome and quality of reporting of descriptive statistics.^12^ Standardized tables for data extraction were employed to gather information on variables such as the lead author, publication year, research methodology, geographic area, rural or urban setting, country of study, size of the sample, data collection techniques, instruments used, and details on suicidal behaviors including their prevalence, incidence, or frequency. A random-effects model was used to synthesize prevalence data, and mixed-effects regression models were employed for subgroup analyses.

### Ethical approval:

507/RC/KEMU; dated 23/05/2024.

## RESULTS

The systematic review aimed to explore trends of suicide in South Asian countries. After rigorous and multistage screening, 27 studies were included for final review. (Table-I) To reduce sampling errors, only those studies included that used random sampling methods. Most of the studies were conducted in one region, but some included more than one country; details are as follows: the study by Howard was conducted in both Bhutan and Nepal; the study by Jordans was conducted in India and Nepal; the study by Nobrega was conducted in Afghanistan, Nepal and Bangladesh; and the study by Smith was conducted in Nepal, Sri Lanka and Bangladesh. The number of studies in different South Asian countries are given in descending order: India (n = 13), Nepal (n = 08), Bhutan (n = 07), Bangladesh (n = 03), Sri Lanka (n = 03), Afghanistan (n = 01); no study was included from Pakistan. Different categories of population were explored; the adult category was included in 11 studies; children were also included in 11 studies; three studies used mixed populations; and one study used older population. Most of the studies were cross-sectional, although one study employed the case control method (Pushpa Kumar) and two studies were prospective longitudinal (Knipe, Benjamin).

**Table-1:** Summary of studies included in systemic Review.

Author Name (Year)	Study Design	Country	Study Population	Method Applied	Study Variables	Focus Area
Abraham et al. (2005).^13^	Observational, Epidemiological	India	Elderly population (≥55 years) in Kaniyambadi block, Tamil Nadu	Verbal autopsy, census data, birth and death records	Suicide rates, age-specific suicide rates, gender differences, suicide methods, demographics (age, gender)	Completed suicides
Armstrong et al. (2013).^14^	Cross-sectional, Community-Based Survey	India	Men who inject drugs (PWID) in Delhi	Time location sampling, structured questionnaire, self-reported symptom scales	Depression, anxiety, suicidal ideation, high-risk behaviors (needle sharing, unprotected sex), HIV risk, demographics (age, marital status, education, income, drug use history)	Suicidal ideation
Arun et al. (2009)^15^	Cross-sectional study	India	School students (Classes VII–XII) in Chandigarh	Random selection of schools, systematic sampling, self-reported questionnaires	Stress, psychological health, suicidal ideation, academic decline, parental influence, demographics (age, gender, family background, education level, socioeconomic status)	Suicidal ideation. Suicidal attempts
Banerjee et al. (2013)^16^	Cross-sectional household survey	India	Households (1680) in the Sundarban region, West Bengal	Mixed random and cluster sampling, structured questionnaire	Deliberate self-harm, suicide rates, pesticide use and storage practices, psychosocial stressors, demographics (age, education, occupation, gender)	Completed suicides, suicidal ideation
Benjamin et al. (2018)^17^	Prospective hospital-based study	India	Patients with non-organophosphate poisoning in a tertiary hospital, South India	Emergency department screening, semi-structured interviews, statistical analysis	Poison type, sociodemographic, poisoning method, impulsivity, premeditation, demographics (age, gender, education, occupation, rural/urban background)	Suicidal attempts
Bhola et al. (2014)^18^	Cross-sectional survey	India	Adolescents (16–18 years) in a pre-university college, Bangalore	Columbia Teen Screen, Strengths and Difficulties Questionnaire (SDQ), structured questionnaires	Suicidal ideation, suicide attempts, emotional and behavioural difficulties, help-seeking behaviour, demographics (age, gender, family structure, education level, socioeconomic status)	Suicidal ideation, suicidal attempts
Bhola et al. (2017)^19^	Cross-sectional study	India	Adolescents and young adults (13–28 years) in urban schools and colleges, South India	Functional Assessment of Self-Mutilation (FASM), Youth Self-Report (YSR), Adult Self-Report (ASR)	Non-suicidal self-injury (NSSI), suicidal intent, internalizing and externalizing problems, predictors of self-injury, demographics (age, gender, family background, education level)	Non-suicidal self-injury (NSSI)
Felez-Nobrega et al. (2020)^20^	Cross-sectional study	48 countries (Afghanistan, Bangladesh, Nepal)	Adolescents aged 12–15 years	Global School-based Student Health Survey (GSHS), logistic regression, meta-analysis	Suicide attempt, physical activity levels, sex differences in suicidality, protective and risk factors, demographics (age, gender, socioeconomic status)	Suicidal attempts
Howard et al. (2021)^21^	Secondary analysis of cross-sectional surveys	Bhutan, Nepal	Secondary school students from Bhutan (n=7576) and Nepal (n=6529)	WHO Global School Health Survey (GSHS), logistic regression, multivariable analysis	Substance use, suicidality, early sexual debut, protective factors, demographics (age, sex, region, parental engagement, school attendance)	Suicidal ideation, suicidal attempts, suicide plans
Islam et al. (2019)^22^	Cross-sectional survey	Bangladesh	Postpartum mothers (15–49 years) in Chandpur District	Structured interviews, multivariate logistic regression	Intimate partner violence (IPV), postpartum suicidal ideation (SI), postpartum depression, self-esteem, demographics (age, education, socioeconomic status)	Suicidal ideation
Jaisoorya et al. (2018)^23^	Cross-sectional study	India (Kerala)	School-going adolescents (n=7560)	Mixed-effect logistic regression, WHO–Thai Health Harm to Others Questionnaire	Alcohol-related harm, psychological distress, suicidality, ADHD symptoms, socioeconomic status	Suicidal ideation, suicidal attempts
Jordans et al. (2018)^24^	Cross-sectional study	Ethiopia, Uganda, South Africa, India, Nepal	Community and primary care populations	Composite International Diagnostic Interview (CIDI) suicidality module, PHQ-9 for depression	Suicidal ideation, suicide attempts, depression, alcohol use disorder, demographics (age, gender, socioeconomic status)	Suicidal ideation, suicidal attempts, suicide plans
Kar et al. (2014)^25^	Cross-sectional, epidemiological survey	India	Tsunami-affected communities of Singarathope village, in the Cuddalore district of Tamil Nadu,	Self-Reporting Questionnaire (SRQ)	PTSD, depression, anxiety, quality of life, disaster exposure, suicidality	Suicidal ideation
Knipe et al. (2019)^26^	Cohort study	Sri Lanka	Left-behind families of migrant workers	Multilevel Poisson regression models, hospital records	Attempted suicide risk, migration, gender differences, demographics (age, gender, socioeconomic status)	Suicidal attempts
Khan et al. (2020)^27^	Cross-sectional study	Bangladesh	School-going adolescents (11–18 years)	Global School-based Student Health Survey (GSHS), Poisson regression	Suicidal ideation, bullying, loneliness, anxiety, peer support, parental monitoring, demographics (age, gender, school environment)	Suicidal ideation
Kumar et al. (2020)^28^	Cross-sectional survey	India	Adolescents in Uttar Pradesh and Bihar	UDAYA project survey	Suicidal tendency, depressive symptoms, demographics	Suicidal ideation
Mamun et al. (2021)^29^	Cross-sectional interview study	Bangladesh	348 flood survivors	Household survey, verbal autopsy	Suicidal behaviour, flood effects, demographics	Suicidal ideation, suicidal attempts, suicide plans
Mashreky et al. (2013)^30^	Cross-sectional study	Bangladesh	819,429 individuals across 12 districts	Household surveys, verbal autopsies	Suicide rates, demographics, methods of suicide	Completed suicides
Neupane et al. (2020)^31^	Cross-sectional study	Nepal	School adolescents (grades 7-11)	Global School-Based Student Health Survey	Bullying victimization, mental health, suicidal behaviour, demographics	Suicidal attempts
Pengpid & Peltzer (2022)^32^	Cross-sectional study	Bhutan	Adults aged 18+ years (national sample)	Bhutan STEPS Survey, Community Survey	Depression, generalized anxiety, socio-demographics, lifestyle behaviors, health status	Suicidal ideation
Poudel et al. (2022)^33^	Cross-sectional study	Nepal	Adolescents (grades 9-12)	Self-administered questionnaires	Non-suicidal self-injury (NSSI), suicidal behaviour, demographics, psychological risk factors	Non-suicidal self-injury, suicidal ideation
Pushpakumara et al. (2021)^34^	Case Control study	Sri Lanka	Self-poisoning cases and matched controls	Structured Clinical Interview for DSM-IV-TR Axis I and II Disorders (SCID I & II)	Deliberate self-poisoning, psychiatric disorders (depression, alcohol use disorder, borderline personality disorder), age, gender, psychiatric morbidity prevalence, health-seeking behavior	Suicidal attempts
Sharma et al. (2019)^35^	Community-based cross-sectional study	India	Ever-married women up to 60 years	In-depth interviews, Self-Reporting Questionnaire (SRQ)	Domestic violence, mental health status, sociodemographic characteristics	Suicidal ideation
Sinha et al. (2021)^36^	Community-based cross-sectional study	India	Adolescents aged 10-19 years	UDAYA project survey	Deliberate self-harm, depressive symptoms, parental abuse, internet access	Non-suicidal self-injury
Smith et al. (2020)^37^	Community-based cross-sectional study	38 countries (Nepal, Bangladesh, Srilanka)	Adolescents aged 12-15 years	Global School-based Student Health Survey (GSHS), CDC Youth Risk Behavior Survey (YRBS)	Sexual behaviour, suicide attempts, age, sex, food insecurity	Suicidal attempts
Thakur et al. (2015)^38^	Cross-sectional study	India	School-going adolescents	Self-administered questionnaire, single item question for suicidal ideation	Suicidal ideation, abuse, family relations, academic performance	Suicidal ideation
Wadood et al. (2021)^39^	Household-based cross-sectional study	Bangladesh	Married adults in Rajshahi City (n=708)	Household suvery, semi-structured interview	Suicidal ideation, suicide attempts, family history, mental comorbidity	Suicidal ideation

Table-II describes Quality assessment done using adapted Newcastle-Ottawa scales for different study types. The quality of the twenty-seven studies was measured across seven matrices: all 27 studies surveyed among populations were representative of the setting, had adequate sample size and used appropriate quality of statistical tests, five studies had adequate response rate, use of valid scales (n=15), presence of sub-group analysis (n=25) and 10 studies used adequate method for assessment of outcome (doctor, researcher or history records). Only two studies scored positively on all criteria. (Table-II) A total of 27 studies were included in the analysis, representing data pertaining to suicidality and related behaviors in South Asian countries. The prevalence of suicide was described as point, period and lifetime prevalence. A multitude of suicidal behaviors were explored, like suicidal thoughts, plans, attempts, completed suicide and non-suicidal behaviors.

**Table-II:** Quality assessment of included studies using adapted Newcastle-Ottawa scales.

Author Name	Representativeness of the Cases	Sample Size	Response Rate	Ascertainment of Scale	Potential Confounders	Assessment of the Outcome	Statistical Tests
		>400=1 <400=0	>95%=1 <95%=0	Diagnostic or valid=2 Non validated=1	Multivariate Analysis/ Subgroup Analysis present=1 Multivariate Analysis Absent=0	Doctor, Researcher, History Records=2 Self-report=1	Appropriate Test done=1 Appropriate Tests not done=0
Abraham et al., 2005	1	1	0	1	0	2	1
Armstrong et al., 2013	1	1	0	1	1	2	1
Arun & Chavan, 2009	1	1	1	2	1	1	1
Banerjee et al., 2013	1	1	1	1	0	1	1
Benjamin et al., 2018	1	1	0	2	1	1	1
Bhola et al., 2013	1	1	0	2	1	1	1
Bhola et al., 2017	1	1	0	2	1	1	1
Felez-Nobrega et al., 2020	1	1	0	1	1	1	1
Howard et al., 2021	1	1	0	1	1	1	1
Islam et al., 2019	1	1	0	2	1	2	1
Jaisoorya et al., 2019	1	1	0	1	1	2	1
Jordans et al., 2018	1	1	0	2	1	2	1
Kar et al., 2014	1	1	0	2	1	1	1
Khan et al., 2020	1	1	0	1	1	1	1
Knipe et al., 2019	1	1	0	2	1	2	1
Kumar et al., 2020	1	1	1	1	1	1	1
Mamun et al., 2021	1	1	1	1	1	2	1
Mashreky et al., 2013	1	1	1	1	1	2	1
Neupane et al., 2020	1	1	0	1	1	1	1
Pengpid & Peltzer, 2021	1	1	1	1	1	1	1
Poudel et al., 2022	1	1	1	2	1	1	1
Pushpakumara et al., 2021	1	1	1	2	1	2	1
Sharma et al., 2020	1	1	1	1	1	1	1
Sinha et al., 2021	1	1	1	1	1	1	1
Smith et al., 2020	1	1	1	1	1	1	1
Thakur et al., 2015	1	1	1	1	1	1	1
Wadood et al., 2021	1	1	0	1	1	2	1

### Point prevalence (Fig.2):

**Fig.2:**
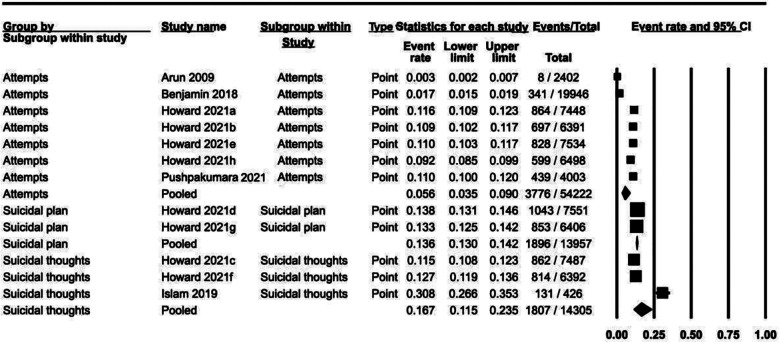
Point prevalence data of suicidal behaviors.

Point prevalence related data was provided for suicidal attempts in seven studies, suicidal plan in two studies and suicidal thoughts in three studies. Using random effects analysis, the prevalence of suicidal thoughts was 16.7% (95% CI: 11.5% to 23.5%), suicidal plan 13.6% (13.0% to 14.2%) and suicidal attempts 5.6% (95% CI: 3.5% - 9.0%). There was significant variability across studies for suicidal attempts (I^2^= 99.52%) and suicidal thoughts (I^2^= 98.35%). Suicidal plan did not show significant heterogeneity (I^2^= 0%).

### Period prevalence (Fig.3):

**Fig.3:**
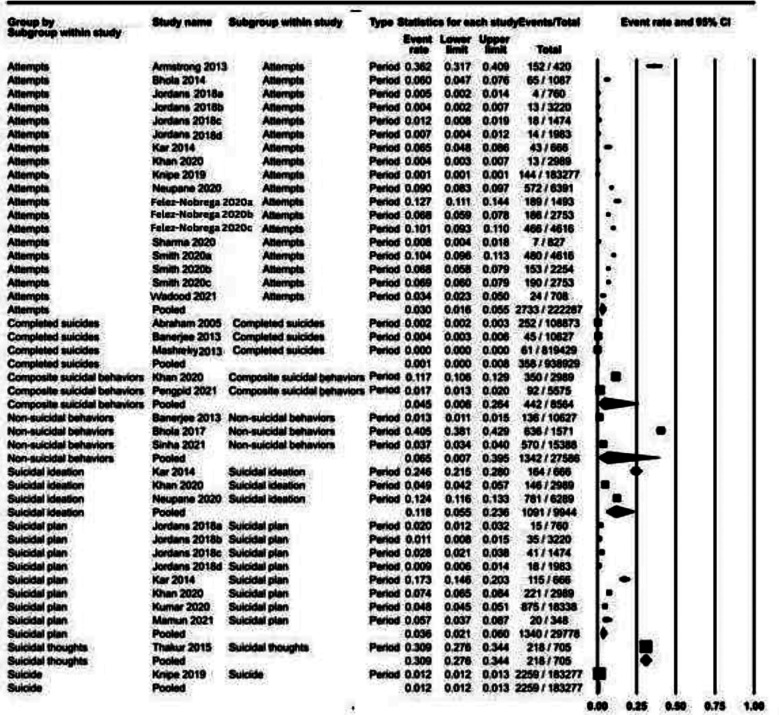
Period prevalence data of suicidal behaviors.

For period prevalence, prevalence for suicidal ideation was noted to be 11.8% (95% CI: 5.5% to 23.6%), plan 3.6% (95% CI: 2.1% to 6.0%). suicidal attempts 3.0% (95% CI: 1.6% to 5.5%), completed suicides 0.1% (95% CI: 0% to 0.8%) and non-suicidal behaviors 6.5% (95% CI: 0.7% to 39.5%). I^2^ for these outcomes ranged from 98.18% to 99.92% for these outcomes. Only suicidal thoughts outcome yielded 0% heterogeneity across studies.

### Lifetime prevalence (Fig.4):

**Fig.4:**
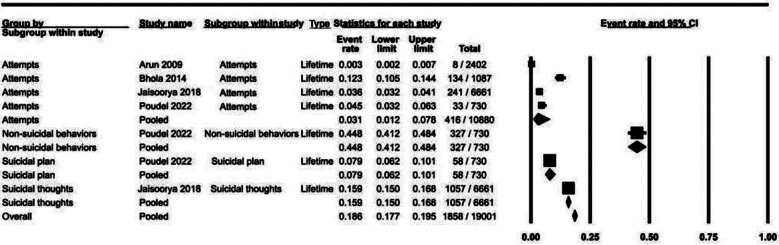
Life time prevalence of suicidal behaviors.

There were seven data points pertaining to lifetime prevalence of suicidal behaviors: attempts (n=4) and one study each for non-suicidal behaviors, plans and thoughts. Suicidal attempts exhibited significant heterogeneity (98.50%). Lifetime prevalence for these studies ranged from suicidal thoughts 15.9% (95% CI:15.0% to 16.8%), suicidal plan 7.9% (95% CI: 6.2% to 10.1%), attempts 3.1% (95% CI: 1.2% to 7.8%) and non-suicidal behaviors 44.8% (95% CI: 41.2% to 48.4%).

## DISCUSSION

Suicidal behaviors despite high burden, remain a low public health priority in South Asia and reliable epidemiological evidence is sparse. In the present study, Suicidal behavior has been estimated in terms of suicidal ideations, suicidal plans and suicidal attempts. The observed pooled prevalence of lifetime suicidal ideation in this study were consistent with the global estimates,^40^ in south Asian women and girls,^8^ as well as students in SEA,^41^ however, our past 12 months suicidal ideation estimates were found to be much higher than observed in European population,^42^ and among students in South Asia.^41^ Compared with other similar studies, the pooled prevalence of lifetime, past 12 months and present attempted suicide is similar to lifetime pooled prevalence of attempted suicide globally and developing countries,^43^ and in Europe.^42^ On the other hand, it is lower than the pooled prevalence among women and girls,^8^ and students in SEA.^41^

A growing body of literature suggests higher risk of suicidal ideation and attempted suicide among women and girls in South Asia. Moreover, comparing prevalence among the population in Europe, China,^44^ and across 17 countries,^42^ we found higher pooled estimates of overall suicidal attempts in South Asia. Our pooled lifetime and past 12 months estimate for suicidal plans are lower than reported in students in South Asia but much higher for the present time.^41^ The lifetime and 12-month prevalence of Non suicidal behaviors in this study were much higher with a high degree of heterogeneity than previously reported globally,^45^ as well as from south Asia.^46^ Differences in socioeconomic status, stigma associated with mental illness, lack of resources and support systems in existing healthcare systems and society’s attitude towards suicidal behaviors affects help seeking and may explain the variations observed in the results.

### Limitations:

First, we considered only peer reviewed journals from selected databases; therefore, articles from non-indexed journals, including articles of unselected databases, were beyond our scope. Several methodological differences across studies contributed to the heterogeneity and the variability of pooled prevalence estimates. Strengths of review included inclusion of studies having more than 400 sample sizes and random sampling only, to ensure adequate methodological quality of the review. To conclude, high prevalence of suicidal behaviors was noted in South Asia. The findings of this review may have several implications for future policy making and practice. Results suggest the need of strengthening the mass awareness campaigns and community screening and surveillance of suicidal behaviors, as well as accessible counselling services especially for high-risk groups. There is need for extensive research to determine cultural factors in suicidal behaviors alongside engaging relevant stakeholders for policy making.

### Authors’ contributions:

**NI and SN:** Conceived the idea of this review article.

**SN, NI, AW, SMT, RKK, MA, BR and HA:** Screened and extracted.

**AW:** Analyzed and interpreted data.

**SN and NI:** Were responsible for the supervision of this project. All authors approved the final version of this review article.

All team members also prepared tables and wrote the manuscript.
